# Identification of eQTLs and sQTLs associated with meat quality in beef

**DOI:** 10.1186/s12864-020-6520-5

**Published:** 2020-01-30

**Authors:** Joel D. Leal-Gutiérrez, Mauricio A. Elzo, Raluca G. Mateescu

**Affiliations:** Department of Animal Sciences, University of Florida, Gainesville, FL USA

**Keywords:** Cis effect, Differentially expressed gene, Expression master regulator, Meat quality, Splicing master regulator and trans effect

## Abstract

**Background:**

Transcription has a substantial genetic control and genetic dissection of gene expression could help us understand the genetic architecture of complex phenotypes such as meat quality in cattle. The objectives of the present research were: 1) to perform eQTL and sQTL mapping analyses for meat quality traits in *longissimus dorsi* muscle; 2) to uncover genes whose expression is influenced by local or distant genetic variation; 3) to identify expression and splicing hot spots; and 4) to uncover genomic regions affecting the expression of multiple genes.

**Results:**

Eighty steers were selected for phenotyping, genotyping and RNA-seq evaluation. A panel of traits related to meat quality was recorded in *longissimus dorsi* muscle. Information on 112,042 SNPs and expression data on 8588 autosomal genes and 87,770 exons from 8467 genes were included in an expression and splicing quantitative trait loci (QTL) mapping (eQTL and sQTL, respectively). A gene, exon and isoform differential expression analysis previously carried out in this population identified 1352 genes, referred to as DEG, as explaining part of the variability associated with meat quality traits. The eQTL and sQTL mapping was performed using a linear regression model in the R package Matrix eQTL. Genotype and year of birth were included as fixed effects, and population structure was accounted for by including as a covariate the first PC from a PCA analysis on genotypic data. The identified QTLs were classified as cis or trans using 1 Mb as the maximum distance between the associated SNP and the gene being analyzed. A total of 8377 eQTLs were identified, including 75.6% trans, 10.4% cis, 12.5% DEG trans and 1.5% DEG cis; while 11,929 sQTLs were uncovered: 66.1% trans, 16.9% DEG trans, 14% cis and 3% DEG cis. Twenty-seven expression master regulators and 13 splicing master regulators were identified and were classified as membrane-associated or cytoskeletal proteins, transcription factors or DNA methylases. These genes could control the expression of other genes through cell signaling or by a direct transcriptional activation/repression mechanism.

**Conclusion:**

In the present analysis, we show that eQTL and sQTL mapping makes possible positional identification of gene and isoform expression regulators.

## Background

Little knowledge exists about transcription variation patterns across the genome as well as how much of this variability is under genetic control. Regulatory variation is proposed as a primary factor associated with phenotypic variability [1] and based on some estimates, gene expression can be classified as medium-highly heritable [2]. Both eQTL and sQTL can be classified into cis (local) and trans (distant) effects. A large fraction of human genes is enriched for cis regulation and in some cases, a cis effect is able to explain trans effects associated with its harboring gene. On the other hand, trans regulation is more difficult to identify and explain [1], but it allows for the identification of “hot spots”, which are also known as master regulators, with transcriptional control over a suite of genes usually involved in the same biological pathway [3]. Therefore, trans regulation might be suggested as the primary factor determining phenotypic variation in complex phenotypes [2].

Since transcription has a substantial genetic control, eQTL and sQTL mapping provides information about genetic variant with modulatory effects on gene expression [4] which are useful for understanding the genetic architecture of complex phenotypes. This mapping allows for uncovering of genomic regions associated with transcription regulation of genes which can be related to phenotypic variation when they colocalize with QTLs (cis and trans effects), providing a molecular basis for the phenotype-genotype association [5]. The eQTL and sQTL mapping can also uncover master regulators and suites of genes related to a particular phenotype (trans effect). Using an eQTL approach, Gonzales-Prendes [6] investigated the genetic regulation of porcine genes associated with uptake, transport, synthesis, and catabolism of lipids. About 30% of these genes were regulated by cis- and/or trans-eQTLs and provided a first description of the genetic regulation of porcine lipid metabolism. Steibel et al. [7] identified 62 unique eQTLs in porcine loin muscle tissue and observed strong evidence for local regulation of lipid metabolism-related genes, such as AKR7A2 and TXNDC12. Higgins et al. [8] carried out an eQTL analysis for residual feed intake, average daily gain and feed intake to identify functional effects of GWAS-identified variants. The eQTL analysis allowed them to identify variants useful both for genomic selection of RFI and for understanding the biology of feed efficiency. Genome sequence-based imputation and association mapping identified a cluster of 17 non-coding variants spanning MGST1 highly associated with milk composition traits [9] in cattle. A subsequent eQTL mapping revealed a strong MGST1 eQTL underpinning these effects and demonstrated the utility of RNA sequence-based association mapping.

The objectives of the present research were: 1) to perform eQTL and sQTL mapping analyses for meat quality traits in *longissimus dorsi* muscle; 2) to uncover genes whose expression is influenced by local or distant genetic variation; 3) to identify expression and splicing hot spots; and 4) to uncover genomic regions affecting the expression of multiple genes (multigenic effects).

## Results

On average, 39.8 million paired-end RNA-Seq reads per sample were available for mapping, and out of these, 34.9 million high-quality paired-end RNA-Seq reads were uniquely mapped to the Btau_4.6.1 reference genome. The mean fragment inner distance was equal to 144 ± 64 bps.

### Expression QTL mapping

**Fig. 1:**
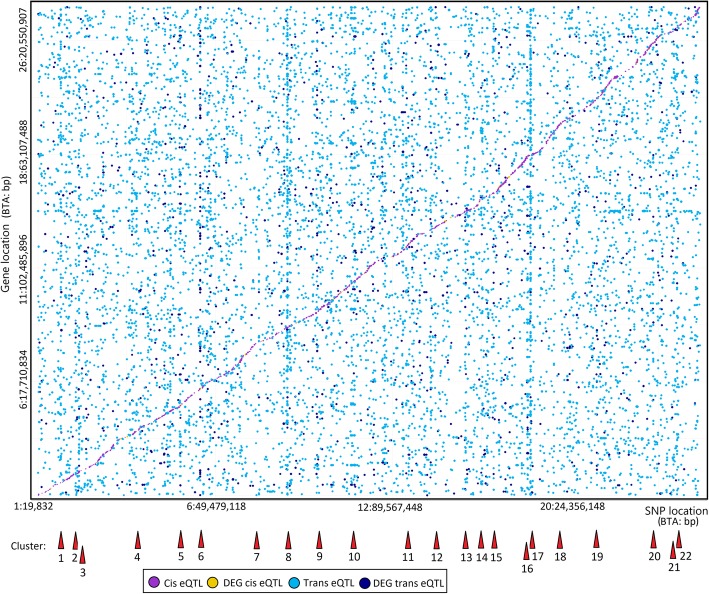
Expression QTL mapping for meat quality in *longissimus dorsi* muscle using 112,042 SNPs and expression data from 8588 genes. A total of 8377 eQTLs were identified. Each dot represents one eQTL and the dot size represents the significance level for each association test. Red triangles locate each cluster of hot spots described in Table 1

**Fig. 2:**
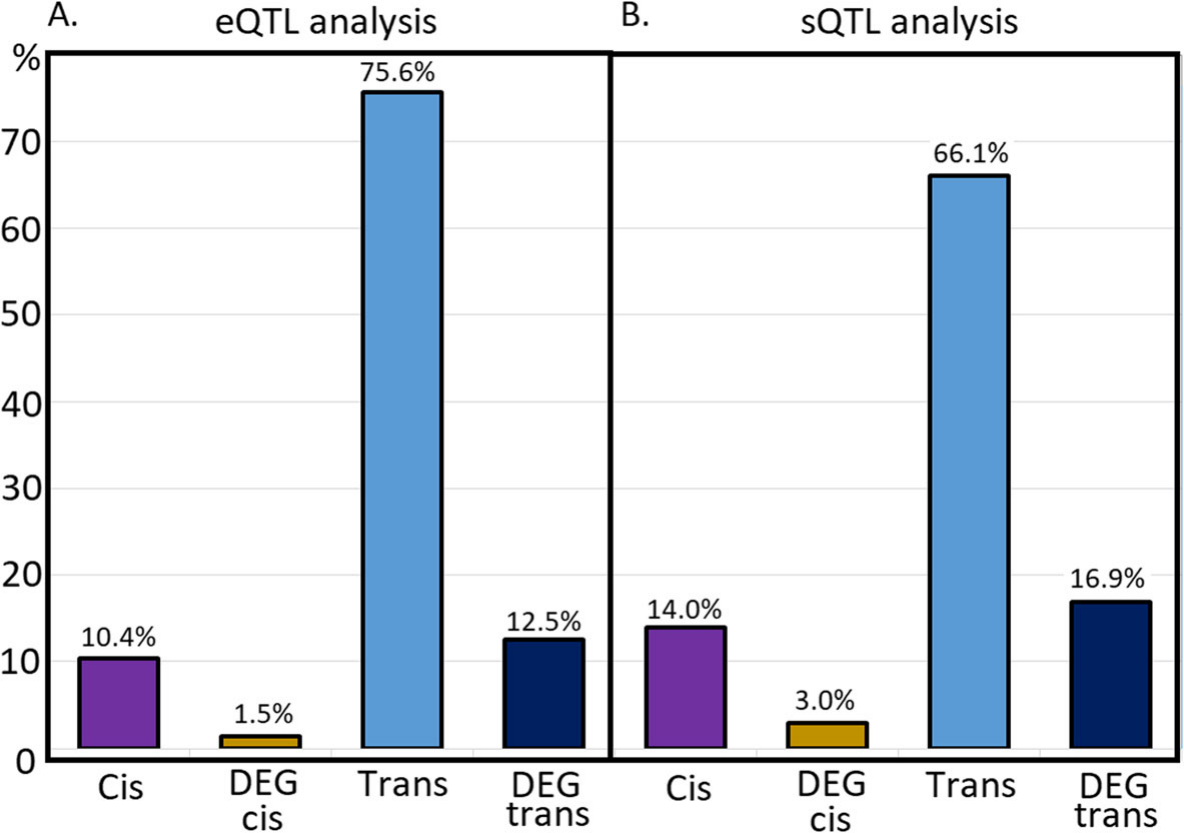
Frequency of each type of eQTL (**a**) and sQTL (**b**) identified. The expression QTL mapping was performed for meat quality related traits in *longissimus dorsi* muscle

A total of 8377 eQTLs were identified in the present population (Fig. 1). The most frequently identified types of eQTLs were trans (75.6%) followed by cis (10.4%) (Fig. 2a). Only 12.5% of the eQTLs were classified as DEG trans and 1.5% as DEG cis. The majority of SNPs with trans and DEG trans effects were associated with the expression of only one gene (76.2 and 84.0%, respectively).

#### Expression cis and DEG cis eQTL analysis

A total of 868 cis and 125 DEG cis eQTLs were uncovered. SNPs rs110591035 and rs456174577 were cis eQTLs and were highly associated with expression of *LSM2 Homolog, U6 Small Nuclear RNA And MRNA Degradation Associated* (*LSM2*) (*p*-value = 5.8 × 10^− 9^) and *Sterol O-Acyltransferase 1* (*SOAT1*) (p-value = 4.4 × 10^− 7^) genes, respectively. Additional file 1 presents all significant eQTLs based on the effective number of independent tests.

#### Expression trans and DEG trans eQTL analysis, and master regulators

**Fig. 3:**
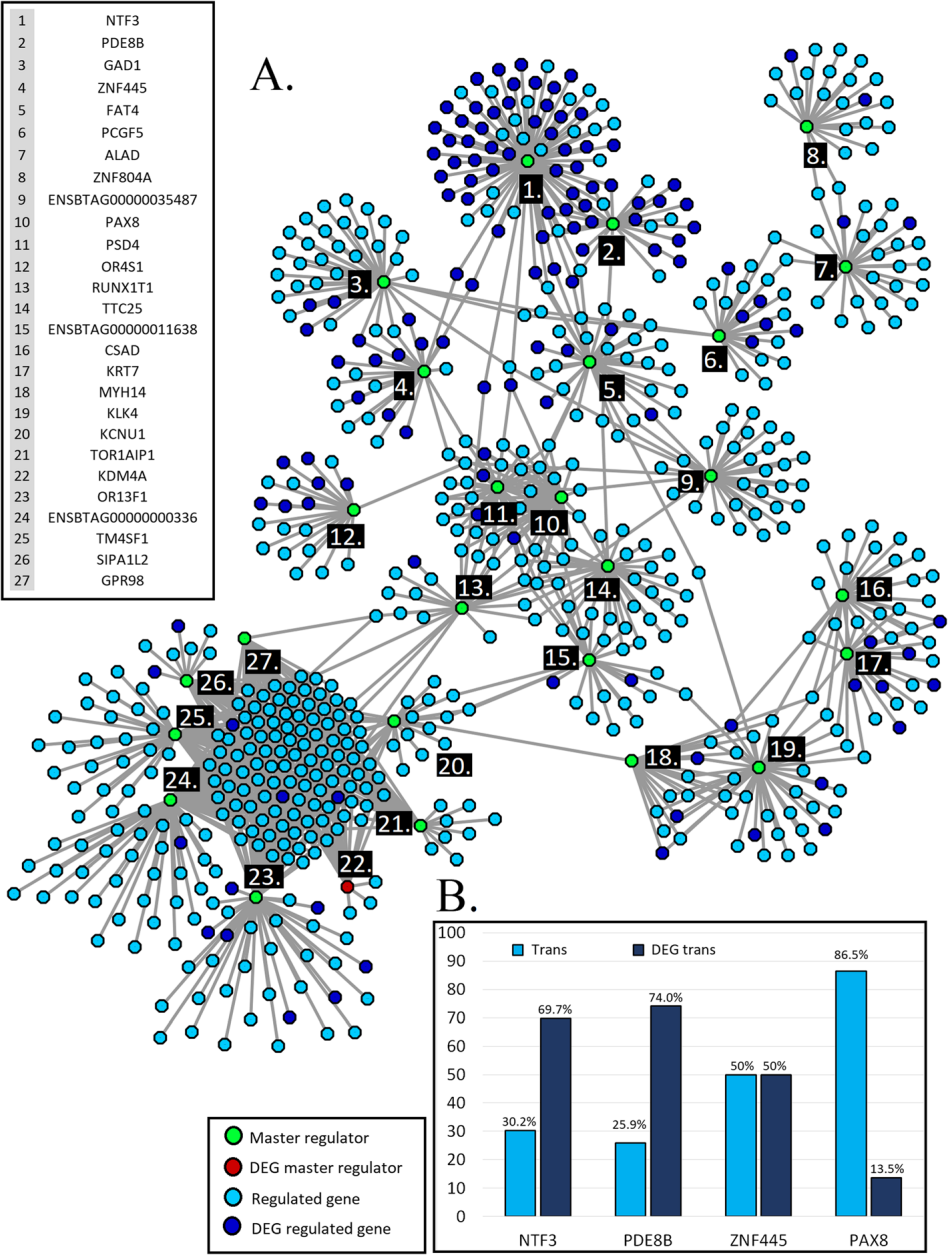
**a** Network of 27 expression master regulators (master regulator in green; differentially expressed master regulator in red) and 674 regulated genes (light blue) or differentially expressed regulated genes identified using eQTL mapping. **b** Percentage of trans and DEG trans regulated genes in the clusters *NTF3*, *PDE8B*, *ZNF445*, and *PAX8*

Twenty-seven SNPs (Table 1) distributed in 22 clusters (Fig. 1) were identified and used to map potential master regulator genes. Figure 3 shows a network for the identified master regulators and their 674 associated genes (Additional file 2). Out of the 27 master regulators, nine membrane-associated proteins, three cytoskeletal proteins, four transcription factors, and one DNA methylase were identified. No clear classification was evident for the remaining 10 genes. Additional file 3 shows least-squares mean plots for SNP effect on transformed gene counts for seven of the identified master regulators.

**Table 1:** Expression QTL master regulators identified in *longissimus dorsi* muscle. The SNP location (BTA: bp), SNP name, cluster number from Fig. 1, minor allele frequency, number of eQTLs associated with each master regulator, the proportion of DEG eQTLs, and the harboring or closest gene are shown for each eQTL master regulator

SNP location	SNP name	Cluster^a^	MAF (%)	Number	% DEG	Harboring gene or closest genes ^b^
				of eQTLs	eQTLs	
1: 119,758,395	rs378343630	1	4	62	0.0	*TM4SF1*
2: 11,594,176	rs208227436	2	3	26	7.7	*ZNF804A*
2: 25,653,736	rs211476449	3	3	36	13.9	*GAD1*
3: 102,943,677	rs135786834	4	2	32	0.0	*KDM4A*
5: 27,001,953	rs441241989	5	8	27	18.5	*CSAD*
5: 27,834,250	rs110130901	5	8	25	24.0	*KRT7*
5: 105,380,442	rs207649022	6	24	76	69.7	***NTF3 -*** *KCNA5*
7: 92,439,344	rs110618957	7	2	34	8.8	*POLR3G -* ***GPR98***
8: 95,625,807	ARS_BFGL_N-GS_65636	8	3	111	8.1	*ENSBTAG00000047350 -* ***OR13F1***
8: 104,345,143	rs378706947	8	2	22	9.1	*ALAD*
10: 8,457,276	Bovine-HD1000002801	9	30	27	74.1	*PDE8B*
11: 46,753,639	rs211218494	10	8	37	13.5	*PSD4*
11: 46,785,388	rs209448226	10	8	37	13.5	*5S_rRNA -* ***PAX8***
13: 54,009,694	rs135144232	11	4	24	8.3	*ENSBTAG00000011638*
14: 74,732,269	rs208451702	12	3	24	8.3	*RUNX1T1*
15: 79,202,054	rs41781450	13	37	20	35.0	*OR4X1 -* ***OR4S1***
15: 79,564,333	rs109630111	13	24	36	2.8	*ENSBTAG00000035487*
16: 62,544,863	rs456174577	14	2	36	0.0	*TOR1AIP1*
17: 30,508,078	BTB_00676236	15	41	34	23.5	*INTU -* ***FAT4***
18: 56,858,212	rs41891374	16	5	20	20.0	*C18H19ORF41 -* ***MYH14***
18: 57,361,426	rs383445569	16	4	41	17.1	*KLK4*
18: 61,257,126	No SNP name	17	49	133	2.3	***ENSBTAG00000000336*** *- ENSBTAG00000046961*
19: 42,754,262	rs377935001	18	2	34	0.0	*TTC25*
22: 16,367,834	rs110289782	19	11	24	50.0	*ENSBTAG00000030533 -* ***ZNF445***
26: 12,930,282	rs42085062	20	4	23	26.1	*PCGF5*
27: 31,921,721	rs136162903	21	3	25	0.0	*KCNU1*
28: 4,877,558	rs207999887	22	5	34	5.9	*SNORA25 -* ***SIPA1L2***

^a^Cluster number used in Fig. 1

^b^Bolded genes were selected as master regulators when the associated SNP was intergenic; underlined gene names were identified as expressed in skeletal muscle in the present analysis.

#### Multigenic effects based on the eQTL analysis

Table 2 shows the number of eQTLs identified by gene where the expression of the top genes seems to be influenced by multiple genomic regions (multigenic effects). The *Solute Carrier Family 43 Member 1* (*SLC43A1*), *Unc-51 Like Autophagy Activating Kinase 2* (*ULK2*), *Myosin Light Chain 1* (*MYL1*), *PHD Finger Protein 14* (*PHF14*), and *Enolase 3* (*ENO3*) are the top five genes based on the number of eQTL regulators.

**Table 2:** Number and type of multigenic effects identified by the eQTL and sQTL analysis performed in *longissimus dorsi* muscle

eQTL analysis			sQTL analysis		
Gene	N	eQTL Type	Gene	N	sQTL Type
*SLC43A1*	126	Trans	*TTN*	324	DEG Trans
*LOC100848703*	64	Trans	*TXN2*	99	Trans
*ULK2*	43	Trans	*NEB*	63	DEG Trans
*MYL1*	40	Trans	*TCEB2*	43	Trans
*ENO3*	36	Trans	*LOC100851645*	36	DEG Trans
*PHF14*	36	Trans	*CREB5*	33	DEG Trans
*PKM*	32	Trans	*USF2*	33	DEG Trans
*ZBTB12*	31	Trans	*MYH7*	28	Trans
*PGAM2*	30	Trans	*PON3*	26	Trans
*ACTA1*	28	Trans	*MYOM3*	26	Trans
*SNX19*	25	Trans	*RSPO2*	25	Trans
*LDHA*	25	Trans	*METTL22*	25	Trans
*RPL5*	23	Trans	*MAP 3 K14*	25	Trans
*ALDH4A1*	23	DEG Trans	*UBR3*	25	Trans
*PLSCR3*	22	Trans	*PAPD4*	25	Trans
*CHURC1*	22	Trans	*BAZ1A*	24	Trans
*TNNI2*	22	Trans	*ITPR1*	23	Trans
*GPD1*	21	Trans	*MYH1*	23	Trans
*TMTC2*	21	Trans	*SVIL*	22	Trans
*UCK2*	21	DEG Trans	*ZDHHC4*	22	Trans
*LRRC42*	20	Trans	*FILIP1L*	22	DEG Trans
			*HSPG2*	21	Trans
			*UBR4*	21	Trans
			*KTN1–2*	21	Trans
			*DST*	21	DEG Trans
			*MYBPC1*	20	Trans

### Splicing QTL mapping

**Fig. 4:**
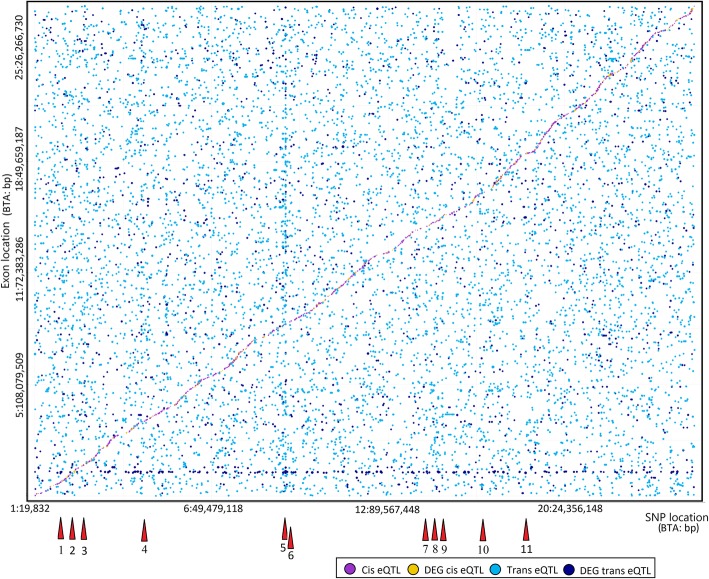
Splicing QTL mapping for meat quality in *longissimus dorsi* muscle using 112,042 SNPs and expression data from 87,770 exons (8467 genes). A total of 11,929 sQTLs were identified. Each dot represents one sQTL and the dot size represents the significance level for each association test. Red triangles show the location of one or several hot spots described in Table 3

The cis and trans sQTLs identified in the present analysis are presented in Fig. 4 and highlight the effects on DEG. A total of 11,929 sQTLs were uncovered. The most frequently identified type of sQTL was trans (Fig. 2b). Trans, DEG trans, cis and DEG cis effects were identified in 66.1, 16.9, 14.0 and 3.0% of the cases, respectively. The majority of SNPs with trans and DEG trans effects were associated with the expression of only one exon (88.4 and 88.9%, respectively).

#### Splicing cis and DEG cis analysis

Additional file 1 shows all cis and DEG cis sQTLs uncovered using the effective number of independent tests. Since the number of significant cis sQTLs detected using these thresholds was very high, only associations with a *p*-value ≤2 × 10^− 4^ were used for further analysis. A total of 2222 cis sQTLs were identified and two of the most interesting genes are *Titin* (*TTN*) and *TEK Receptor Tyrosine Kinase* (*TEK*).

#### Splicing trans and DEG trans sQTL analysis, and master regulators

Out of the 13 splicing master regulator genes identified in the present analysis (Table 3), four encode for proteins located in the extracellular space. Four other genes encode for plasma and/or organelle associated membrane or cytoskeletal proteins, and two other genes encode for transcription factors. Mechanisms associated with splicing regulation for the remaining three master regulators were not evident. A total of 231 genes (Additional file 4) were associated with these 13 master regulators and were included in a regulation network (Additional file 5). The master regulators *ZNF804A*, *ALAD, OR13F1,* and *ENSBTAG00000000336* were determined simultaneously as expression and splicing master regulators. Markers inside these four genes were able to explain variability in the fraction of exon counts in 28 (*ZNF804A*), 192 (*ALAD*), 22 (*OR13F1*) and 25 (*ENSBTAG00000000336*) genes across the genome. The most important uncovered master regulators associated with splicing were selected for further discussion.

**Table 3:** Splicing QTL master regulators identified in *longissimus dorsi* muscle. The SNP location (BTA: bp), SNP name, cluster number from Fig. 4, minor allele frequency (MAF), number of sQTLs associated with each master regulator, the proportion of DEG sQTLs, and the harboring or closest gene are shown for each eQTL master regulator

SNP location	SNP name	Cluster^a^	MAF (%)	Number	% DEG	Harboring gene or closest genes^b^
				of sQTLs	sQTLs	
1: 144,604,558	rs381222773	1	7	33	9.1	***PDE9A*** *-* *WDR4*
2: 11,594,176	rs208227436	2	3	28	17.9	*ZNF804A*
2: 84,792,003	rs208053623	3	4	21	19.0	*DNAH7*
4: 5,827,343	rs381476620	4	42	21	33.3	*ZPBP* *-* ***VWC2***
8: 92,924,658	rs382101207	5	3	23	13.0	*RNF20*
8: 93,336,078	BTB_01634267	5	4	20	20.0	*PLEKHB2* *-* ***SNORA19***
8: 95,762,113	rs136343964	5	3	22	13.6	*OR13F1*
8: 104,345,143	rs378706947	6	2	192	27.1	*ALAD*
14: 57,184,022	rs210798753	7	2	24	50.0	*PKHD1L1*
15: 25,536,733	rs209617551	8	2	34	35.3	*SNORA3*
15: 35,729,304	rs382501844	9	4	33	9.1	***NUCB2*** *- ENSBTAG00000032859*
16: 75,296,157	rs41821837	10	4	20	20.0	***SYT14*** *-* *DIEXF*
18: 61,257,126		11	49	25	44.0	***ENSBTAG00000000336*** *- ENSBTAG00000046961*

^a^Cluster number used in Fig. 4

^b^Bolded genes were selected as master regulators when the associated SNP was intergenic; underlined gene names were identified as expressed in skeletal muscle in the present analysis

Two different clusters were uncovered in the Functional Annotation Clustering analysis using the whole list of regulated genes across clusters (Additional file 6). Some of the identified terms in these clusters were Carbon metabolism, ATP binding and Nucleotide-binding, showing that genes in these clusters might have a complex splicing regulation.

#### Multigenic effects based on the sQTL analysis

A variety of genes seem to have a complex transcriptional control based on the ratio of exon counts (Table 2) and some of them are: *Titin* (*TTN*), *Nebulin* (*NEB*), *Elongin B* (*TCEB2*), *CAMP Responsive Element Binding Protein 5* (*CREB5*) and *Upstream Transcription Factor 2, C-Fos Interacting* (*USF2*).

## Discussion

### Expression QTL mapping

#### Expression cis and DEG cis eQTL analysis

*LSM2* and *SOAT1* harbor some highly significant cis eQTLs. LSM2 binds to other members of the ubiquitous and multifunctional family Sm-like (LSM) in order to form RNA-processing complexes. These complexes are involved in processes such as stabilization of the spliceosomal U6 snRNA, mRNA decay and guide site-specific pseudouridylation of rRNA [10]. Lu et al. [11] identified two missense polymorphisms in *SOAT1* associated with cholesterol in plasma and triglyceride levels in mice since they are able to increase enzyme activityG. None of these two genes were identified as DEG, therefore they must be more involved in skeletal muscle homeostasis.

#### Expression trans and DEG trans eQTL analysis, and master regulators

The 27 master regulators identified in the eQTL analysis could contribute to gene expression control by promoting cell signaling or by direct transcriptional activation/repression mechanisms. A number of structural proteins and transcription regulators were identified as master regulators. *Neurotrophin 3* (*NTF3*), *Glutamate Decarboxylase 1* (*GAD1*), *FAT Atypical Cadherin 4* (*FAT4*), *Transmembrane 4 L Six Family Member 1* (*TM4SF1*), *Transmembrane 4 L Six Family Member 1* (*TM4SF1*) and *Keratin 7* (*KRT7*) encode for transmembrane or cytoskeletal proteins. *Zinc Finger Protein 804A* (*ZNF804A*), *Paired Box 8* (*PAX8*), *Lysine Demethylase 4A* (*KDM4A*) and *RUNX1 Translocation Partner 1* (*RUNX1T1* or *Myeloid Translocation Gene on 8q22-MTG8*) encode for transcription factors or histone demethylases. *NTF3*, *TM4SF1,* and *KDM4A* are further discussed.

*NTF3* was identified as a master regulator in the present analysis since rs207649022 was able to explain variation in the expression of 76 genes (Table 1), 69.7% of which were DEG genes (Fig. 3b). Since *NTF3* was associated with a number of DEGs, this master regulator was able to explain variability in gene expression associated with meat quality. The Neurotrophic Factor gene family regulates myoblast and muscle fiber differentiation. It also coordinates muscle innervation and functional differentiation of neuromuscular junctions [12]. Mice with only one functional copy of the *NTF3* gene showed a smaller cross-sectional fiber area and more densely distributed muscle fibers [13]. Upregulation of *NTF3*, stimulated by the transcription factor POU3F2, is present during neuronal differentiation [14]. The neocortex has multiple layers originated by cell fate restriction of cortical progenitors and NTF3 induces cell fate switches by controlling a feedback signal between postmitotic neurons and progenitors. Therefore, changes in *NTF3* expression can modulate the amount of tissue present in each neocortex layer [15].

*NTF3* was identified in a previous study as highly associated with cooking loss [16] pointing out that markers inside this locus are able to explain variation at both the phenotype and gene expression level. This implicates *NTF3* as a positional and functional gene with a potential role in meat quality. These effects are probably not due to cis regulation on *NTF3* given that the number of reads mapped to this gene was extremely low and it did not surpass the threshold used in order to be included in the DEG analysis (average = 6.7, min = 0; max = 23). However, *NTF3* could be actively expressed in earlier developmental stages and then expressed at a basal level, exerting control on expression regulation later on when cellular morphology has been completely established. A Functional Annotation Clustering analysis for the *NTF3* regulated genes indicated that the master regulator *NTF3* could be involved in the regulation of specific mechanisms and pathways related to Mitochondrion, Transit peptide and Mitochondrion inner membrane (Additional file 6).

The expression of 62 genes was associated with rs378343630, a marker located in the *TM4SF1* master regulator. This gene encodes a plasma transmembrane protein and belongs to a gene family involved in signal transduction processes; thus, it modulates development, growth, and motility [17]. TM4SF1 physically interacts with the membrane and some cytoskeleton-associated proteins to form cell projections named ‘nanopodia’ [18], which are described as frequently identified in multiple types of cancer. This gene is highly expressed in pancreatic cancer cells and stimulates metastasis by upregulating *Discoidin Domain Receptor Tyrosine Kinase 1* (*DDR1*), *Matrix Metallopeptidase 2* (*MMP2*) and *Matrix Metallopeptidase 9* (*MMP9*) [19]. In liver, TM4SF1 reduced apoptosis and promoted cell migration by upregulating *MMP-2*, *MMP-9* and *VEGF*, and downregulating *Caspase-3* and *Caspase-9* [17]. Upregulation of *miR-9* produces downregulation of *TM4SF1*, *MMP2*, *MMP9* and *VEGF* in colorectal carcinoma, inhibiting cell migration and invasion [20]. In esophageal cancer stem-like cells, downregulation of *miR-141* increases *TM4SF1* expression, self-renewal ability and promotes cell invasion [21]. The Functional Annotation Clustering analysis for *TM4SF1* found an overrepresentation of the transcription, DNA-templated term (Additional file 6); thus, *TM4SF1* could be involved in the regulation of specific mechanisms and pathways associated with transcription in *longissimus dorsi* muscle. Neither *TM4SF1* nor any gene in this cluster was identified as DEG; therefore they might be more related to skeletal muscle homeostasis than meat quality.

The *KDM4A* cluster has 32 regulated genes associated with rs135786834; *KDM4A* encodes a histone lysine demethylase able to modify trimethylated H3-K9/K36 to dimethylated products, contributing to gene expression, cellular differentiation and cancer development [22]. Histone H3K9 methylation promotes the silencing of muscle-specific genes in proliferating myoblasts and derepression of these genes is required to initiate muscle differentiation. Expression of a *KDM4A* isoform named DN-JMJD2A is upregulated during differentiation of myoblasts into myotubes promoting myotube formation and transcriptionally activating muscle-specific genes such as *MyoD* [23]. The only DEG master regulator identified in the present analysis was *KDM4A* and this master regulator harbors rs135786834, an SNP associated with expression of 32 genes by trans association. Therefore, changes in the expression of *KDM4A* did not show evidence of promoting the expression of genes related to meat quality.

#### Multigenic effects based on the eQTL analysis

Some of the most interesting genes identified in this analysis were *ULK2*, *MYL1,* and *PHF14*. Forty-three SNPs were associated with *ULK2* expression. *ULK2* encodes a serine/threonine-protein kinase required for autophagy under nutrient-deprived conditions [24]. Downregulation of *ULK2* activates mTOR c1 signaling, promoting cell proliferation [25]. The *MYL1* gene encodes a fast-twitch regulatory light chain of myosin in skeletal muscle; downregulation of *MYL1* alters myocyte morphology and muscle structure, and generates congenital myopathy in zebrafish [26]. A total of 40 and 36 polymorphisms were associated with the expression of *MYL1* and *PHF14*, respectively. *PHF14* is ubiquitously expressed and its protein has multiple PHD fingers, a domain present in chromatin-binding proteins which are able to recognize particular epigenetic marks on histone tails. The *PHF14* knockout in mice generates neonatal lethality and severe structural changes in multiple organs especially lungs. PHF14 is an epigenetic regulator required for the normal development of multiple organs [27], and it is probably involved in skeletal muscle homeostasis.

### Splicing QTL mapping

#### Splicing cis and DEG cis analysis

The *TTN* gene harbors a highly significant DEG cis sQTL (*p*-value = 2.0 × 10^− 7^) and encodes a central sarcomeric protein. Some *TTN* mutations are associated with skeletal-muscle diseases such as tibial muscular dystrophy [28]. Fernandez-Marmiesse et al. [29] identified a non-sense mutation in a *TTN* exon only present in a fetal skeletal isoform and associated with a neuromuscular disorder; histologically, this mutation promotes sarcomeric deposition of a filamentous material. A DEG cis sQTL (*p*-value = 5.1 × 10^− 7^) was identified in the *TEK* gene. This gene encodes a receptor for *Angiopoietin-1* (*ANGPT1*), and its signaling pathway is critical for migration, sprouting and survival of endothelial cells; TEK activates the SHC Adaptor Protein 1 (SHC1), a protein involved in triggering the Ras/mitogen-activated protein kinase pathway, regulating migration and endothelial organization [30]. Therefore, cis sQTLs in *TTN* and *TEK* could explain variation in the expression of these genes and variation in meat quality-related phenotypes.

#### Trans and DEG trans splicing QTL analysis, and master regulators

Similarly, as the identified expression master regulators, the splicing master regulators can be grouped as transcription regulators and structural proteins. *Small Nucleolar RNA, H/ACA Box 3A* (*SNORA3*), *Small Nucleolar RNA, H/ACA Box 19* (*SNORA19*), *Ring Finger Protein 20* (*RNF20*), and *Zinc Finger Protein 804A* (*ZNF804A*) could be classified as transcription regulators. *Phosphodiesterase 9A* (*PDE9A*), *Olfactory Receptor Family 13 Subfamily F Member* (*OR13F1*), *Dynein Axonemal Heavy Chain 7* (*DNAH7*) and *Von Willebrand Factor C Domain Containing 2* (*VWC2*) can be identified as structural proteins.

Small non-coding RNAs such as *SNORA3* and *SNORA19* modulate stability, folding and interaction with proteins and more recently, functions such as mRNA editing, alternative splicing and posttranscriptional gene silencing were discovered [31]. However, no clear function of *SNORA3* and *SNORA19* is described. Exon expression of 34 exons from 17 genes and 20 exons from 15 genes were associated with the polymorphisms rs209617551 (*SNORA3*) and BTB_01634267 (*SNORA19*), respectively.

Expression of 23 exons from 19 genes was associated with rs382101207, an SNP located in *Ring Finger Protein 20* (*RNF20*). Upregulation of *RNF20* stimulates H2B monoubiquitination and methylation at H3K4 and H3K79; it promotes expression of Homeobox genes, a group of transcription factors [32]. *RNF20* also regulates expression of *H2A* and *H2B* histones, *p53*, several proto-oncogenes and promotes cell migration and tumorigenesis [33]. The *RNF20*/*RNF20* (Bre1 complex) is documented as a tumor suppressor by upregulating a set of tumor suppressor genes and by contributing to genomic stability maintenance. Bre1 deficient cells present a high frequency of DNA double-strand breaks (DSB), and abundant aberrant RNA-DNA structures (R-loops), indicators of replication stress and genomic instability [32].

Pierce et al. [1] theorized that a high proportion of trans associations are caused by cis effects. However, no cis QTL was identified in any expression or splicing master regulator. This result suggests that, in the present analysis, trans effects might contribute significantly to phenotypic variation related to skeletal muscle homeostasis and meat quality.

#### Multigenic effects based on the sQTL analysis

The large number of sQTLs identified in genes like *TTN (324)* and *NEB* (63) could be related to gene size, since these genes are 275 and 219 kb long, respectively, which would increase the probability of being involved in trans regulation. On the other hand, some relatively short genes such as *TCEB2* (9.9 kb) and *USF2* (3.9 kb) also had a large number of sQTLs (43 and 33, respectively) indicating a possible complex splicing regulation.

A total of 324 and 67 polymorphisms were associated with *TTN* and *NEB* ratio exon counts, respectively. TTN and NEB are involved in assembly and mechanical activity of striated muscles. Both proteins are large sarcomere filament-binding proteins expressed in skeletal muscle and multiple splicing events in the bovine homologous are described. In the human brain, NEB acts as an actin filament stabilizer and regulates neuronal length. It is also involved in myofibrillogenesis, modulates thin filament length and allows proper muscle contraction [34]. *TTN, NEB,* and *USF2* were identified as DEG; therefore, sQTL regulation could contribute to phenotypic variability associated with meat quality in *longissimus dorsi* and skeletal muscle homeostasis.

### Gene expression and splicing regulation mechanisms by plasma and organelle associated proteins

The cell cytoskeleton provides cellular mechanical constraints and extracellular matrix stiffness [35]. However, structural proteins are involved in multiple biological processes different from the organizational ones, with signaling and cell fate being some of the most important. Cell signaling is crucial since it orchestrates cellular responses to different microenvironmental stimuli, and transcription repression-activation and splicing regulation are influenced by signaling proteins. A number of receptors, transmembrane linkers, cytoskeletal fibers and membrane-associated transcription factors were previously associated with transcription repression-activation.

The *OR4A47*, *GPR98*, *PDE9A*, *OR13F1* and *SYT14* master regulators were also described as transmembrane protein-coding genes and this type of molecule is involved in cell signaling processes. Pandey et al. [36] reported that estrogen can signal using diverse receptors, the G Protein-Coupled Estrogen Receptor 1 (GPR30) being one of them. Stimulation of GPR30 by estrogen activates a transcription factor network that upregulates *Cellular Communication Network Factor 2* (*CCN2*), promoting proliferation and cell migration. The master regulators *GAD1* and *TM4SF1* encode transmembrane linkers similar to the integrin family. Integrins can modulate signal transduction cascades involved in cell survival, proliferation, differentiation and organ development [37]. The dimer ITGA1-ITGB1 can stall Epidermal Growth Factor Receptor (EGFR) signaling by stimulating Protein Tyrosine Phosphatase, Non-Receptor Type 2 (PTPN2). The cytoplasmic domain of ITGA1 interacts with PTPN2 and decreases EGFR phosphorylation after Epidermal Growth Factor (EGF) stimulation [38].

The cytoskeletal protein-coding genes *KRT7*, *FAT4*, *MYH14,* and *DNAH7* were identified as master regulators. Some cytoskeletal proteins might drive transcription regulation and promote cellular mechanisms such as growth and apoptosis. Flouriot et al. [35] reported that the actin network can regulate Myocardin Related Transcription Factor A (MRTFA) subcellular localization, a protein involved in growth-quiescence switch. High F/G actin ratio or mutant MRTFA cells showed higher global biosynthetic activity and open chromatin state, associated with extensive histone modifications. In Drosophila, Hippo tumor suppressor pathway controls organ size, and proteins such as Yorkie (human homologous *Yes Associated Protein 1* -*YAP*), a transcriptional coactivator, and Hpo and Warts kinases (human homologous *Serine/Threonine Kinase 3* -*STK3*- and *Large Tumor Suppressor Kinase 1* -*LATS1*, respectively) belong to this pathway. YAP is negatively regulated by STK3 and LATS1. F-actin accumulation promotes overgrowth in Drosophila imaginal discs through modulating the activity of the Hippo pathway [39].

### Applicability of the present results and future analysis

The present results provide biological support to some of the previously identified pQTLs related to complex phenotypes in cattle and could contribute to discovery of potential causative polymorphisms. pQTL and eQTL colocalization for *NTF3* (cooking loss) and *GPR98* (tenderness) was evident in the present population [16]; however, more research is required in order to be able to determine if these genes harbor actual causative markers associated with meat quality. The use of causative polymorphisms in genomic prediction is the ideal scenario since it is not affected by recombination events between the actual pQTL and the marker being genotyped, over time. In this respect, research showed that polymorphisms associated with expression regulation such as eQTLs and sQTLs can explain an important proportion of the genetic variance present on complex phenotypes in cattle.

Lopdell et al. [4] identified a set of 3695 distinct eQTL variants for milk, fat and protein yield and showed that they have increased the predictive ability for milk composition related phenotypes. *DGAT1*, *MGST1,* and *GPAT4* were identified as the most highly predictive regions. A 1 Mbp region nearby *DGAT1* harbors three polymorphisms that are able to explain a high amount of the SNP variance in the set. Xiang et al. [40] classified 17,669,372 imputed variants into 30 sets of markers. This classification included categories such as inter-species conserved markers, polymorphisms associated with metabolic traits (several milk metabolites), expression regulation related polymorphisms (gene and exon expression QTLs, sQTLs, and allele-specific expression QTLs), and markers with evolutionary roles. An index was constructed for each marker using the amount of genetic variance explained by them for a total of 34 complex traits in cattle. Conserved markers, polymorphisms associated with metabolic traits and expression regulation related markers were able to explain the highest amount of genetic variance. Later, this index was applied to another population composed of 7551 individuals and it was determined that high ranking variants significantly increased genetic variance estimates and genomic prediction accuracies for milk, fat and protein yield.

However, other research has found difficult to illustrate the potential use of eQTL and sQTL mapping on the predictive ability for complex phenotypes. The research of Berg et al. [41] was focused on identifying pQTLs caused by eQTL for milk, fat and protein yield, and calving interval. No strong evidence of association between pQTL and eQTL effects were evident.

The results reported by Berg et al. [41] could indicate that most eQTLs are able to explain a very small fraction of the variance associated to pQTLs; however, it is important to highlight that lack of power for eQTL effect estimation and long-range LD could contribute the difficulty of identifying pQTLs and eQTL colocalization. Additionally, the relationship between pQTL and eQTL effect could be dependent on the genetic architecture of the phenotype being assessed and its degree of transcriptional control. In this respect, Lopdell et al., [4] noticed that predictions for milk, fat and protein yield using eQTL variants did not surpass R^2^, of 0.5 since all the QTL effects present in these traits are not due to expression effects. Furthermore, eQTLs in related tissues or eQTLs present at different stages of development could contribute to these phenotypes as well.

In order to identify causative polymorphisms, the present results require validation through eQTL and sQTL mapping on additional populations with Angus, Brahman and mixed breed composition. After validation, candidate genes would also need to be confirmed using in-vitro and in-vivo analysis. For the assessment of proteins described as eQTL and sQTL associated transcription factors, techniques such as Electrophoretic mobility shift assay (EMSA) and Chip-seq could be used in order to identify actual DNA-protein interaction able to regulate gene expression of the potential target genes. To support eQTL and sQTL master regulator activity for structural proteins able to activate signaling cascades and gene expression, knockout and knockdown trials could verify if these proteins could module this biological activity. Finally, to identified cis regulations, reporter gene experiments can be used.

## Conclusions

The mapping analysis performed in this study provides a holistic insight into the regulatory network architecture in *longissimus dorsi* muscle in an Angus-Brahman population.

Multiple cis eQTLs and sQTLs effects were identified and genes such as *LSM2*, *SOAT1*, *TTN* and *TEK* are a few examples of potential expression and splicing regulatory genes. A total of 27 expression and 13 splicing master regulator genes were uncovered, mainly cytoskeletal or membrane-associated proteins, transcription factors and DNA methylases. Cytoskeletal proteins provide mechanical constraints to the cell, but they are also involved in processes such as signaling. Signaling is crucial since it coordinates cellular responses to different stimuli, and transcription repression-activation and splicing regulation are influenced by structural proteins. The *ZNF804A*, *ALAD, OR13F1* and *ENSBTAG00000000336* genes were identified as both expression and splicing master regulators.

It is shown that eQTL and sQTL mapping makes possible positional identification of potential expression and splicing master regulators. The present analysis identified master regulators associated with gene and isoform expression in skeletal muscle but was also focused on uncovering master regulators related to genes whose expression is able to explain variability in meat quality-related phenotypes (DEG genes) in cattle. The genes *PDE8B*, *NTF3*, *ZNF445,* and *OR4S1* can be highlighted as eQTL master regulators associated with a high proportion of DEG genes. The sQTL master regulators *PKHD1L1*, *ENSBTAG00000000336*, *SNORA3,* and *VWC2* were the regulators most frequently associated with DEG genes. These master regulators could contribute to phenotypic variability through modulating the expression of key genes whose expression is able to explain variability in the complex meat quality phenotype.

## Methods

### Cattle population and phenotypic data

The University of Florida Institutional Animal Care and Use Committee number 201003744 approved the present research protocol. A total of 120 steers from the University of Florida Beef Unit multibreed Angus-Brahman herd born between 2013 and 2014 were used in this study [42]. This population can be classified into six different groups based on breed composition. In terms of Angus composition, the grouping was the following: 1 = 100 to 80%; 2 = 79 to 65%; 3 = 64 to 60% (Brangus); 4 = 59 to 40%; 5 = 39 to 20%; 6 = 19 to 0% [42].

These animals were kept with their dams on bahiagrass pastures (*Paspalum notatum*) until weaning and received a complete mineral supplement (UF University Special Hi-Cu Mineral, University of Florida, Gainesville, Florida), and bermudagrass (*Cynodon dactylon*) hay and cotton-seed (Gossypium spp.) meal in the winter months (mid-December to mid-March). The calves were kept on bahiagrass pastures and fed bahiagrass hay, concentrate (1.6–3.6 kg of soy hull pellets per day; 14.0% CP; 488 Pellet Medicated Weaning Ration, Lakeland Animal Nutrition, Lakeland, Florida) and a mineral supplement until yearling.

Yearling steers were transported to a contract feeder (2014: Suwannee Farms, O Brien, Florida; 2015: Quincey Farms, Chiefland, Florida), where they were provided a standard feedlot diet based on corn, protein, vitamins, and minerals until they reached a subcutaneous fat thickness over the ribeye of approximately 1.27 cm [43]. Cattle were transported to a commercial processing facility (FPL Food LLC., Augusta, Georgia) 1 day prior to harvest. Steers were harvested under USDA-FSIS inspection using captive bolt stun. The average slaughter weight was 573.34 ± 54.79 kg at 12.91 ± 8.69 months. After splitting the carcass, five to ten g of the *longissimus dorsi* muscle was collected, snapped-frozen in liquid nitrogen and stored at − 80 °C until RNA was extracted.

Phenotypes recorded on these steers included tenderness, connective tissue and juiciness determined by a sensory panel, and marbling, cooking loss and WBSF according to the American Meat Science Association Sensory Guidelines [44]. Marbling was assessed on the ribeye muscle at the 12th/13th rib interface after ribbing the carcass and was recorded on a numerical scale by visual appraisal 48 h *postmortem*. The grading was as follows: Practically Devoid = 100–199, Traces = 200–299, Slight = 300–399, Small = 400–499, Modest = 500–599, Moderate = 600–699, Slightly Abundant = 700–799, Moderately Abundant = 800–899, Abundant = 900–999.

From each animal, two 2.54 cm steaks from the 12th/13th rib interface of the *longissimus dorsi* muscle were collected, aged for 14 days at 4 °C, and stored at − 20 °C at the Meat Science Laboratory of the University of Florida. Frozen steaks were allowed to thaw at 4 °C for 24 h and cooked to an internal temperature of 71 °C on an open-hearth grill.

After cooking, the first steak was cooled at 4 °C for 18 to 24 h and six cores with a 1.27-cm diameter and parallel to the muscle fiber were sheared with a Warner-Bratzler head attached to an Instron Universal Testing Machine (model 3343; Instron Corporation, Canton, MA). The Warner-Bratzler head moved at a cross head speed of 200 mm/min. The average peak load (kg) of six cores from the same animal was analyzed. The weight lost during cooking was recorded and cooking loss was expressed as a percentage of the cooked weight out of the thaw weight. The second steak was cooked and assessed by the sensory panel. The sensory panel consisted of eight to 11 trained members, and six animals were assessed per session. Two 1 × 2.54 cm samples from each steak were provided to each panelist. Sensory panel measurements analyzed by the sensory panelists included: tenderness (8 = extremely tender, 7 = very tender, 6 = moderately tender, 5 = slightly tender, 4 = slightly tough, 3 = moderately tough, 2 = very tough, 1 = extremely tough), juiciness (8 = extremely juicy, 7 = very juicy, 6 = moderately juicy, 5 = slightly juicy, 4 = slightly dry, 3 = moderately dry, 2 = very dry, 1 = extremely dry), and connective tissue (8 = none detected, 7 = practically none, 6 = traces amount, 5 = slight amount, 4 = moderate amount, 3 = slightly abundant, 2 = moderately abundant, 1 = abundant amount). For each phenotype, the average score by steak from all members of the panel was analyzed.

Marbling, WBSF, cooking loss, juiciness, tenderness and connective tissue were included in a principal component (PC) analysis using PROC FACTOR procedure of SAS [45], and a composited meat quality index for each animal was constructed using the first three PCs. The meat quality index was determined using the following formula:

$$ Meat\ {quality\ index}_i=\sum \limits_{j=1}^3\left({PCS}_{ij}\ast {PCW}_j\right) $$

Where PCS_ij_ is the PC score of the animal i for the PC_j_ and PCW_j_ is the weight of the PC_j_ (eigenvalue). The amount of variance explained by PC_1_, PC_2_ and PC_3_ were 44.26, 20.04 and 13.29%, respectively. The 120 animals were ranked using the meat quality index and 80 animals with extreme values were selected and used for RNA sequencing.

### Genotyping and data quality control

Genomic DNA was extracted from blood using the DNeasy Blood & Tissue kit (Qiagen, Valencia, CA) and stored at − 20 °C. All animals were genotyped with the commercial GGP Bovine F-250 chip (GeneSeek, Inc., Lincoln, NE) which contains 221,077 single nucleotide polymorphisms (SNPs). After excluding markers with a minor allele frequency lower than 3% (at least 2 animals out of 80 for the less frequent genotype) and a calling rate < 0.9, a total of 112,042 SNPs were included in the association analysis. Quality control was implemented with JMP genomics 6.0 software [46]. The genotype data is available in the European Variation Archive website, accession number PRJEB24746.

### RNA extraction, RNA-seq library preparation and sequencing

Total RNA was extracted from muscle using TRIzol reagent (Thermo Fisher Scientific, Waltham, MA, USA) according to the manufacturer’s protocol (Invitrogen, catalog no. 15596–026). RNA concentration was measured by NanoDrop 2000 spectrophotometer (Thermo Fisher Scientific, Waltham, MA, USA) and integrity was verified by formaldehyde gel. The mRNA samples were stored at − 80 °C. Total RNA samples were sent to RAPiD Genomics LLC (Gainesville, Florida, United States) for mRNA isolation, RNA-seq library preparation and sequencing procedures. mRNA isolation was performed using oligo-dT attached magnetic beads prior to its reverse transcription and synthesis of double-stranded cDNA. One RNA-seq library for each sample was constructed, multiplexed, and sequenced based on protocols of Illumina HiSeq 3000 PE100 platform (Illumina, San Diego, CA, USA). All samples were sequenced on 8 lanes, generating 2 × 101 nts paired-end reads. RNA-seq data are available at the European Nucleotide Archive, accession number PRJEB31379.

### Read quality control, paired-end read alignment and paired-end read counting

The pipeline described by Korpelainen et al. (2014) [47] was used to generate an index for the Btau_4.6.1 reference genome and to create a gene, exon and isoform expression files. Tophat 2.1.0 [48], Bowtie2 2.3.4 [49], Picard [50] and samtools [51] were used to generate the Btau_4.6.1 index. Eight forward and eight reverse FASTQ files per sample were concatenated in separated FASTQ files and analyzed with FastQC 0.9.6 [52] to check quality of the raw sequence reads. Read trimming was performed with PRINSEQ 0.20.4 software [53] using 3 bp sliding windows and 20 as phred threshold. Reads with more than 2 ambiguous bases were excluded from the analysis. Cutadapt version 1.8.1 software [54] was used to trim adapters and reads shorter than 50 nts were excluded.

Tophat 2.1.0 [48] and Bowtie2 2.3.4 [49] were used to perform paired-end read mapping against the Btau_4.6.1 reference genome [55]. HTSeq 0.9.1 software [56] was used to estimate gene paired-end read counts for all annotated genes, including paired-end reads uniquely mapped to known chromosomes. Cufflinks 2.2.1.1 [57, 58] was used to assemble transcripts and estimate transcript abundance in FPKM (Fragments Per Kilobase of exon per Million fragments mapped). Exon counts per gene were determined using the RNA-Sequencing differential expression analysis pipeline DEXSeq [59]. Genes and exons with less than 10 counts across all 80 samples were excluded from the analysis. Indexing and sorting of the alignment files were performed using Samtools 1.9 software [51].

### Differentially expressed genes, exons and isoforms associated with meat quality

Differential expression analysis was performed to identify genes, exons and isoforms whose expression was associated with meat quality. The procedures described by Seo et al. [60], Love et al. [61] and Jia et al. [62] were used to identify differential expression. Genes and exons with less than 10 counts, and isoforms with less than 10 FPKM across samples were excluded from the analysis.

The R package edgeR [63] was used to obtain normalized gene counts by applying the trimmed mean of M-values (TMM) normalization method. The R packages sfsmisc and MASS [64–66] were used to apply Huber’s M-estimator based robust regression including all 80 samples used for RNA sequencing. The meat quality index was used as a response variable. Gene expression was treated as a covariate and year of birth of the animal as fixed effects. A PCA analysis was carried out with the “PCA for population structure” work-flow of JMP [46], and population structure was accounted for by including its first PC as covariate in the model. Genes whose association test had a *p*-value lower than 0.05 were included in the DEG list. The same analysis was performed for exon expression, and genes with at least three associated exons were included in the DEG list.

Out of the 80 samples used for RNA sequencing, 40 (20 high and 20 low performance based on WBSF, tenderness or marbling) were included in the DEG analysis. The R package DESeq2 version 1.20.0 [61] was used to identify DEG genes, including year of birth, breed group and a categorical classification of each animal based on phenotype as fixed effects in the analysis. The categorical classification was as follows: tender vs tough using WBSF or tenderness and high vs low using marbling. Genes with a Benjamini-Hochberg adjusted p-value lower than 0.05 were determined as DEG for WBSF and lower than 0.1 as DEG for tenderness and marbling. The DEG isoform analysis was performed with MetaDiff [62]. Breed group, year of birth, and the same categorical classification based on phenotype fitted in the DESeq2 analysis were included as fixed effects in the association model.

A total of 8799 genes, 93,349 exons, and 4471 isoforms from 957 genes were included in the DEG analysis. Expression of 1352 genes was identified as associated with meat quality traits using the differential expression analysis (Additional file 7).

### eQTL and sQTL mapping

The R package Matrix eQTL was used to perform the QTL mapping [67] using 112,042 SNPs and 8588 genes (eQTL mapping) or 87,770 exons from 8467 genes (sQTL mapping) located in autosomes. A linear regression model was used where the SNP genotypes were coded as 0, 1 or 2. For the eQTL analysis, gene counts were transformed using the tool varianceStabilizingTransformation from the R package DESeq2 version 1.20.0 [61] in order to solve heteroscedasticity [8]. In the sQTL analysis, we used the fraction of counts mapped to a specific exon out of the total counts mapped to its gene [68]. This fraction was converted to an integer value by keeping three decimal digits and multiplying by 1000 and then transformed using the tool varianceStabilizingTransformation. Gene and fraction exon counts were included as response variables, and SNP genotype and year of birth of the animal as fixed effects. The first PC from the “PCA for population structure” work-flow of JMP [46] was included as a covariate in the model to control for population structure. A cis QTL was defined as an SNP located no more than 1 Mb upstream of the transcription start site or downstream of the transcription end site of an annotated gene, and cis and trans QTLs were analyzed separately.

Bonferroni trans and cis *p*-value thresholds were calculated using the effective number of independent tests implemented in the R function “simpleM_Ex” [69]. For the trans associations, the total number of tests was 112,042, and 42,246 was its corresponding effective number of independent tests. Therefore, the p-value corrected for multiple testing for the trans effects was equal to 1.18 × 10^− 6^ for both, trans eQTLs and sQTLs. However, in order to maximize the number of eQTLs and sQTLs hotspots a less stringent threshold was used. The final trans association thresholds used for eQTLs and sQTLs were 1 × 10^− 5^ and 1 × 10^− 6^, respectively. An effective number of independent tests per each gene was calculated in order to determine cis p-value thresholds. An in-house script written in Java was used to group all SNPs by gene and to generate the file inputs for the R function “simpleM_Ex” [70]. The Bonferroni cis p-value thresholds are presented in the Additional file 8. However, since the number of cis sQTLs was very high using these thresholds, a more stringent threshold was implemented. The final cis sQTL association threshold was 2 × 10^− 4^.

Polymorphisms associated with expression of at least 20 genes in the case of eQTL and at least 20 exons in the case of sQTL were considered hot spots. The harboring gene or the adjacent gene in which biological function was somewhat related to transcription regulation was defined as master regulators.

### Functional annotation clustering analysis

A functional classification analysis using DAVID Bioinformatic Resources 6.8 server [71] was performed for each cluster of genes associated with a master regulator.

## Supplementary information

**Additional file 1. **Cis eQTL and sQTL effects identified in *longissimus dorsi* muscle sampled from a multibreed Angus-Brahman herd. SNP information about 112,042 markers and expression data from 8588 genes and 87,770 exons from 8467 genes were included in the association assay. Out of the 8588 genes included, expression of 1352 loci was previously identified as related to meat quality traits and denoted as DEG genes.

**Additional file 2. **List of 674 regulated genes and their respective expression master regulator identified by the eQTL association mapping. The eQTL mapping was performed in *longissimus dorsi* muscle sampled from a multibreed Angus-Brahman herd. SNP information about 112,042 markers and expression data from 8588 genes were included in the association assay.

**Additional file 3. **Least-square mean plots for SNP effect on transformed gene counts of some regulated genes. Genes regulated by the master regulators *TM4SF1* (rs378343630), *GAD1* (rs211476449), *PCGF5* (rs42085062), *RUNX1T1* (rs208451702), *KLK4* (rs383445569), *CSAD* (rs441241989) and *OR4S1* (rs41781450) are shown.

**Additional file 4. **List of 231 regulated genes and their respective splicing master regulators identified by the sQTL association mapping. The sQTL mapping was performed in *longissimus dorsi* muscle sampled from a multibreed Angus-Brahman herd. SNP information about 112,042 markers and expression data from 87,770 exons (8467 genes) were included in the association assay

**Additional file 5. 4A.** Network for 13 splicing master regulators and 231 regulated genes identified using sQTL mapping. **4B.** Percentage of trans and DEG trans regulated genes in the clusters ALAD, PKHD1L1 and SNORA3. Network for 13 splicing master regulators and percentage of trans and DEG trans regulated genes

**Additional file 6. **Enriched terms identified by DAVID Bioinformatic Resources 6.8 server functional classification analysis. These terms were found enriched when performing analysis inside each expression or splicing master regulator cluster, or across regulated genes. The QTL mapping was performed in *longissimus dorsi* muscle sampled from a multibreed Angus-Brahman herd.

**Additional file 7. **List of DEG and expressed genes. A list of 1352 genes considered as DEG genes for meat quality in the present analysis, and 8799 genes identified as expressed in *longissimus dorsi* muscle in a multibreed Angus-Brahman herd.

**Additional file 8. **Bonferroni cis thresholds by gene. The number of tests, number of effective tests and the Bonferroni adjusted *p*-value is presented by each gene used in the association analysis.

## Data Availability

Genotype data are available on the EVA website, accession number PRJEB24746. https://www.ebi.ac.uk/ena/data/view/PRJEB24746. RNA-seq data are available at the European Nucleotide Archive, accession number PRJEB31379, https://www.ebi.ac.uk/ena/data/search?query=PRJEB31379.

## Abbreviations

DEG: Differentially expressed gene
eQTL: Expression quantitative trait loci
pQTL: Phenotypic quantitative trait loci
SNP: Single nucleotide polymorphism
sQTL: Splicing quantitative trait loci
