# Supplementary figures and images for: Identification of eQTLs and sQTLs associated with meat quality in beef
Source: BMC Genomics. 2020 Jan 30;21:104. doi: 10.1186/s12864-020-6520-5 (PMC6993519; doi:10.1186/s12864-020-6520-5)

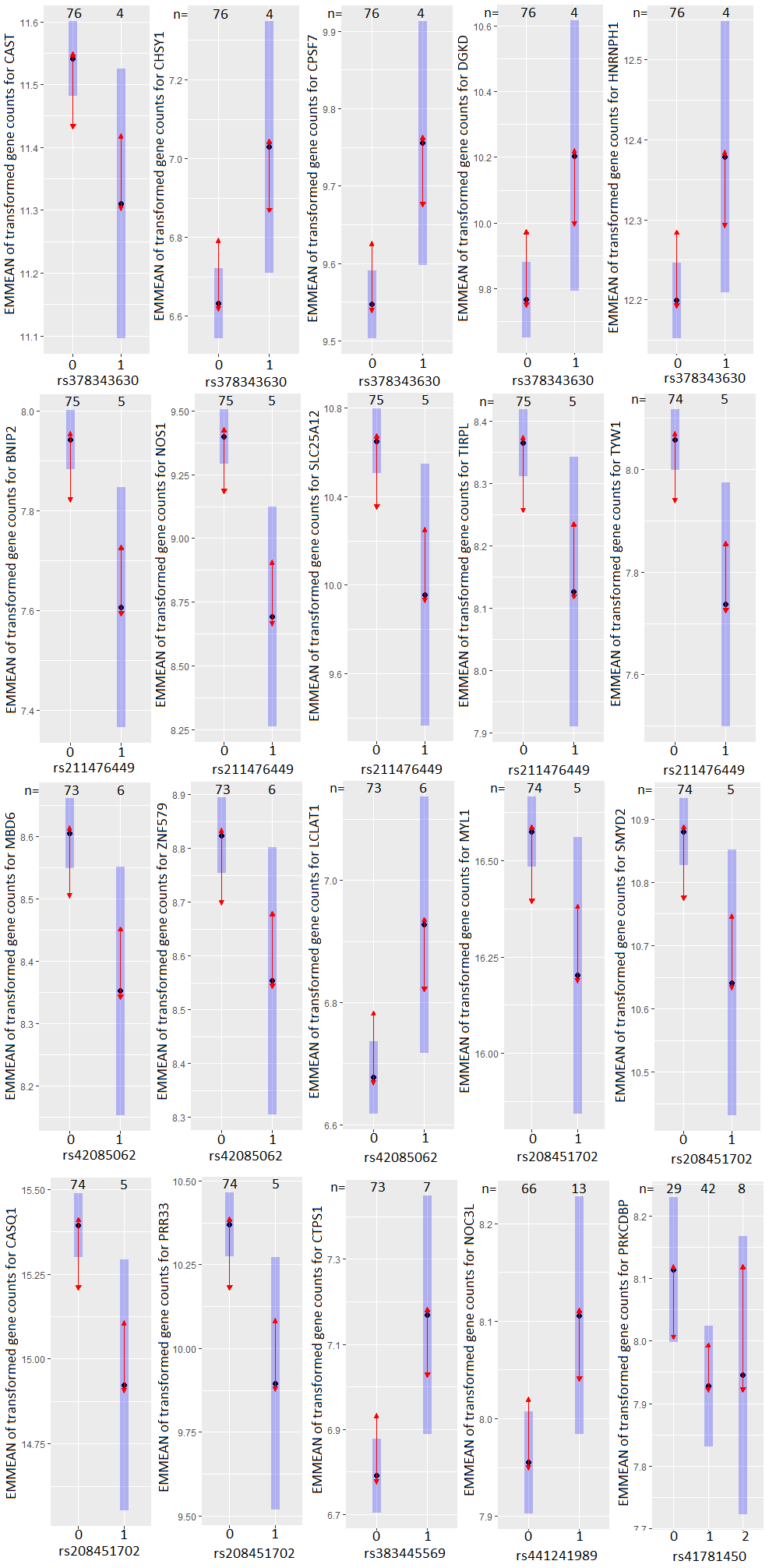

Supplement: Supplementary file 3 — Additional file 3. Least-square mean plots for SNP effect on transformed gene counts of some regulated genes. Genes regulated by the master regulators TM4SF1 (rs378343630), GAD1 (rs211476449), PCGF5 (rs42085062), RUNX1T1 (rs208451702), KLK4 (rs383445569), CSAD (rs441241989) and OR4S1 (rs41781450) are shown. [file 12864_2020_6520_MOESM3_ESM.png]

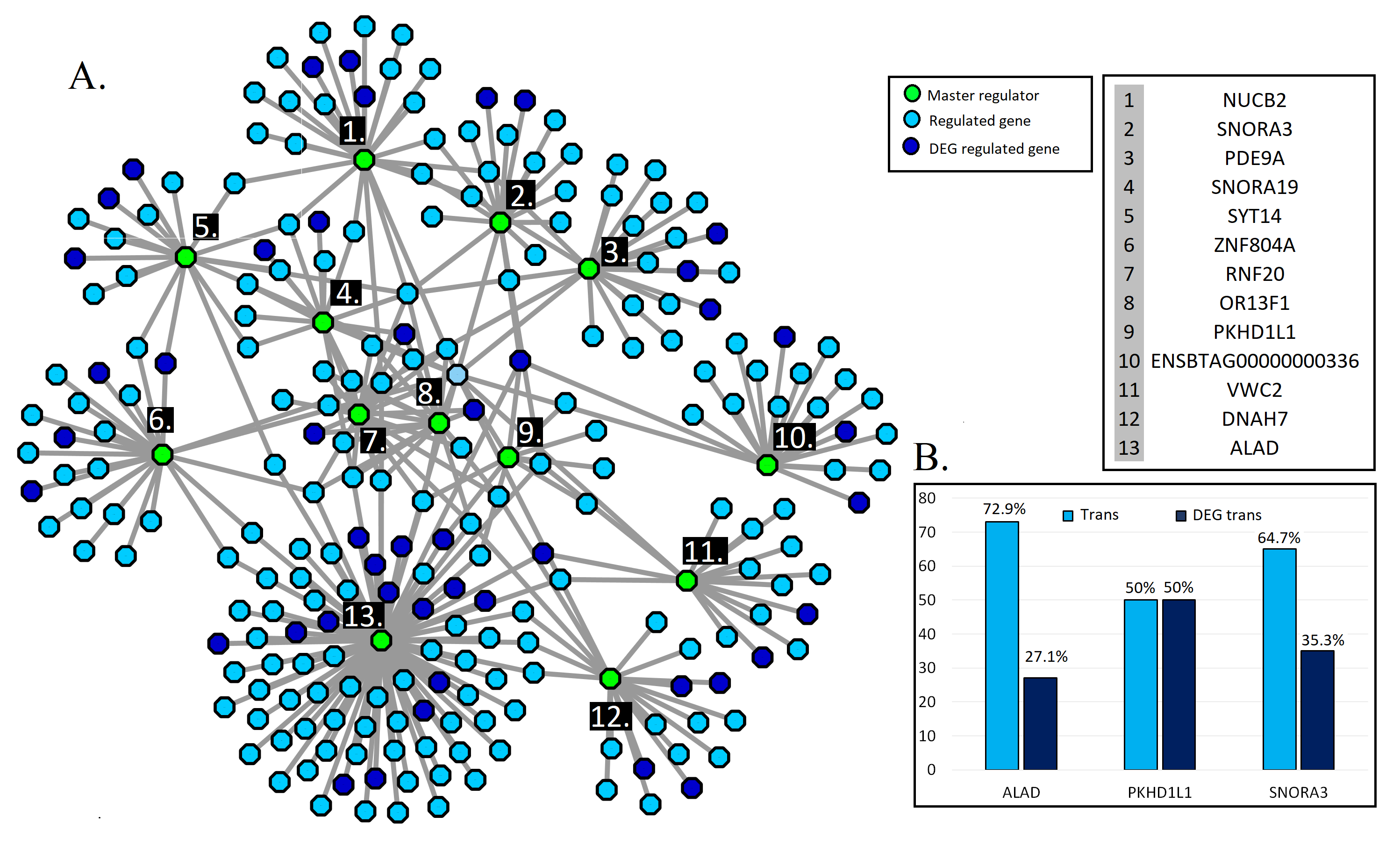

Supplement: Supplementary file 5 — Additional file 5. 4A. Network for 13 splicing master regulators and 231 regulated genes identified using sQTL mapping. 4B. Percentage of trans and DEG trans regulated genes in the clusters ALAD, PKHD1L1 and SNORA3. Network for 13 splicing master regulators and percentage of trans and DEG trans regulated genes [file 12864_2020_6520_MOESM5_ESM.png]
